# Development of Modular and Adaptive Laboratory Set-Up for Neuroergonomic and Human-Robot Interaction Research

**DOI:** 10.3389/fnbot.2022.863637

**Published:** 2022-05-11

**Authors:** Marija Savković, Carlo Caiazzo, Marko Djapan, Arso M. Vukićević, Miloš Pušica, Ivan Mačužić

**Affiliations:** ^1^Faculty of Engineering, University of Kragujevac, Kragujevac, Serbia; ^2^mBrainTrain d.o.o., Belgrade, Serbia; ^3^School of Food Science and Environmental Health, Technological University Dublin, Dublin, Ireland

**Keywords:** modular and adaptive laboratory workstation, experimental set-up, cognitive ergonomics, human-robot collaboration, Poka—Yoke system, musculoskeletal disorders, industry−4.0

## Abstract

The industry increasingly insists on academic cooperation to solve the identified problems such as workers' performance, wellbeing, job satisfaction, and injuries. It causes an unsafe and unpleasant working environment that directly impacts the quality of the product, workers' productivity, and effectiveness. This study aimed to give a specialized solution for tests and explore possible solutions to the given problem in neuroergonomics and human–robot interaction. The designed modular and adaptive laboratory model of the industrial assembly workstation represents the laboratory infrastructure for conducting advanced research in the field of ergonomics, neuroergonomics, and human–robot interaction. It meets the operator's anatomical, anthropometric, physiological, and biomechanical characteristics. Comparing standard, ergonomic, guided, and collaborative work will be possible based on workstation construction and integrated elements. These possibilities allow the industry to try, analyze, and get answers for an identified problem, the condition, habits, and behavior of operators in the workplace. The set-up includes a workstation with an industry work chair, a Poka–Yoke system, adequate lighting, an audio 5.0 system, containers with parts and tools, EEG devices (a cap and smartfones), an EMG device, touchscreen PC screen, and collaborative robot. The first phase of the neuroergonomic study was performed according to the most common industry tasks defined as manual, monotonous, and repetitive activities. Participants have a task to assemble the developed prototype model of an industrial product using prepared parts and elements, and instructed by the installed touchscreen PC. In the beginning, the participant gets all the necessary information about the experiment and gets 15 min of practice. After the introductory part, the EEG device is mounted and prepared for recording. The experiment starts with relaxing music for 5 min. The whole experiment lasts two sessions per 60 min each, with a 15 min break between the sessions. Based on the first experiments, it is possible to develop, construct, and conduct complex experiments for industrial purposes to improve the physical, cognitive, and organizational aspects and increase workers' productivity, efficiency, and effectiveness. It has highlighted the possibility of applying modular and adaptive ergonomic research laboratory experimental set-up to transform standard workplaces into the workplaces of the future.

## Introduction

Numerous studies and research articles show that integrating innovative advanced technologies of Industry 4.0 utilizing lean and ergonomic helps to enhance the health and safety of the workers performing monotonous, manual, repetitive, physical demanding assembly activities at the workstations in contemporary organizations (Schwab, 2016; Battini et al., 2020; Pinzone et al., 2020) and to increase the efficiency of the operators by improving performance, reducing production time, and reducing errors (Colim et al., 2021).

With the increasing customer demand for unique, customized, personalized, low-cost products in small batches in the shortest possible time, organizations are being pressurized to proactively answer and to improve the flexibility and effectiveness of the production systems to maintain a competitive advantage in the market (Battini et al., 2011; Battaïa et al., 2018). The abovementioned can be achieved through automation and manufacturing advancement (Tsarouchi et al., 2016; El Zaatari et al., 2019), introducing collaborative robots and other innovative Industry 4.0 technologies into production processes (Tobe, 2015; Salunkhe et al., 2019; Cimini et al., 2020).

The monotonous, repetitive movements at high speed at the industrial workstations are often performed in ergonomically inadequate and non-physiological body positions over a long period. It can cause occupational diseases (Shikdar and Garbie, 2011) such as mental and physical effort (Schaub et al., 2013), fatigue, discomfort, forearm muscle effort, extreme joint positions, which increases the risk of back pain and musculoskeletal disorders (Barr et al., 2004) and other health and safety problems (Petreanu and Seracin, 2017).

In the European Union member states, musculoskeletal disorders (MSD) are one of the leading health problems of workers (Maurice et al., 2017), causing absenteeism, inefficiency, and productivity loss in the manufacturing industry (Schneider et al., 2010; Bevan, 2015; El Makrini et al., 2019). MSD arises from repetitive movements of body parts, awkward postures (Ranavolo et al., 2020), high demand for work or low autonomy, and low job satisfaction (Petreanu and Seracin, 2017). The installation of EMG sensors enables monitoring of muscle activity during the assembly activities of parts and components and determines the load and tension of the neck, arm, and shoulder muscles during these activities. In this way, it is determined that when the first symptoms of MSD begin to appear, the frequency of pain in different regions of the body is examined so that appropriate preventive measures could be taken (Segning et al., 2021).

Some research suggests a link between conditions in which workers perform uncomfortable activities and decreased productivity (Liao and Drury, 2000; Dainoff, 2002; Haynes and Williams, 2008; Husemann et al., 2009). Numerous scientific research articles indicate the importance of an ergonomically acceptable designed work environment where repetitive assembly work is performed (Coury et al., 2000; Isa et al., 2011). In that case, special attention must be paid to the “golden zone” (Sanders and McCormick, 1993). This zone is the cylindrical segment-shaped area from the worker's waist to shoulder height and with forearm length as the radius. As the golden zone is different for each worker, the workstation ensures that workspace and arrangement of materials, components, and tools positions could be adapted to the individual needs. Also, human–robot collaborative interaction has been proposed as a potential solution to improve workplace conditions, eliminate risk factors, and improve wellbeing and satisfaction through physical and cognitive aspects need to be considered (Fast-Berglund et al., 2016; Kadir et al., 2018; El Zaatari et al., 2019; Prati et al., 2021).

At industrial workstations where manual, repetitive, and assembly activities are performed, human errors are almost inevitable, and numerous errors cannot be easily detected at the further stages of production or during inspection (Wallace and Vodanovich, 2003). Timely detection of falls in attention and concentration through advanced EEG research contributes to improving Occupational Safety and Health (OSH)—reducing injuries during work and reducing accidents that could be fatal in some situations (Parasuraman and Rizzo, 2006; Strasser, 2021; Botti et al., 2022.

The motivation for writing this scientific research article could be found in the fact that MSD, ergonomics, and neuroergonomics have many common points that should be identified and researched in the future within scientific research. Examining the mental and emotional reactions, monitoring operators' performance, and examining all significant factors that affect them during the cooperation between collaborative robots and workers is an open question that should be explored in the future through scientific research. Researching the behavior of operators, monitoring neuroergonomics parameters during collaborative work, and monitoring attention and fatigue contribute to a better understanding of the phenomena that occur and indicate the specifics of workers' behavior. To achieve the above, it is possible to design and develop a modular and adaptive ergonomic research laboratory experimental set-up for human–robot interaction and to test it according to the already defined scenarios.

## Literature Review

Konz (1995) and Das (2007) pointed out that job creation with non-respect for the ergonomic principles is common in the industry. Concerning this, performing complex operations of assembling parts and components in non-ergonomic postures on the workstations is an essential field of research for many researchers (Loch et al., 2016). Performing activities in an ergonomically inadequate workplace can cause MSDs, physical and emotional stress on the workers, low efficiency and productivity, and unsatisfactory product quality (Ulin and Keyserling, 2004). Chiasson and Major (2015) surveyed 473 workers in 1 year. The examination results showed that a large percentage of workers had MSDs and that a large number of workers reported feeling pain. Bernal et al. (2015) consider that MSD is more conditioned by psychological and social risk factors than physical factors.

Numerous studies and research articles have shown that long-term work in a sitting position results in increased feelings of discomfort for the workers (McLean et al., 2001; Fenety and Walker, 2002; Callaghan et al., 2010). Some authors believe that the most significant discomfort in the lower extremities occurs when workers perform activities only in a standing position (Roelofs and Straker, 2002). Frequently, changes in the body position and performing activities combined with sitting and standing positions and increasing breaks reduce discomfort (McLean et al., 2001).

Scientific literature showed that ergonomic intervention is the best strategy to improve workers' health and safety by preventing MSD and reducing injuries during the work, discomfort, absenteeism (Burdorf, 2010; Takala et al., 2010; Botti et al., 2014), and enhancing operator performance, productivity, efficiency, product quality, and reliability (Hendrick, 2003; Dul et al., 2004; Roper and Yeh, 2007; Vayvay and Erdinc, 2008; Neumann and Dul, 2010). Furthermore, law regulations in this area remind organizations of the importance of including an ergonomic aspect when designing a prefabricated workstation (Otto and Scholl, 2011). The authors have proved that the application of ergonomic principles in the workplace directly impacts reducing errors and increasing product quality (Jorgen and Eklund, 1995; Hamrol et al., 2011; Thun et al., 2011; Falck and Rosenqvist, 2012). Yeow and Sen (2006) believe that even the cheapest ergonomic solutions can significantly have a positive effect on the quality of activities. González et al. (2003) showed in their study that product quality increased by 2% and additional processing of the finished product was significantly reduced after the improvement of physical ergonomics. Previous studies on improving assembly performance have focused mainly on conducting a batch experiment of different products, optimal distribution of the activities, including assembly activities (Arnold et al., 2004; Ullah et al., 2009).

In particular, some authors pointed out the importance of developing fully adjustable and ergonomically designed innovative workstations compared with the non-ergonomically designed fixed traditional workstations (Eswaramoorthi et al., 2010) to perform repetitive assembly tasks (Temple and Adams, 2000; Shikdar and Hadhrami, 2007). Other authors pointed out the advantages of performing workstation activities in an adequate ergonomic position, minimizing worker movements during the working activities (Roelofs and Straker, 2002; Lin and Chan, 2007; Davis et al., 2009). According to Muhundhan (2013), placing materials, parts, and tools at operators' fingertips reduces unnecessary stretching reach and, in that way, worker's fatigue is also reduced.

The design of the workstation can be facilitated by the innovative technologies of Industry 4.0 (Burggräf et al., 2019). Some studies showed the digital transformation of the manual workstation into a collaborative one (Pini et al., 2016; Gualtieri et al., 2020; Colim et al., 2021; Palomba et al., 2021) and indicated the benefits of collaborative cooperation between operators and robots (Consiglio et al., 2007; Sadrfaridpour and Wang, 2017; Heydaryan et al., 2018; Castro et al., 2019; Liau and Ryu, 2020; Parra et al., 2020; Pérez et al., 2020). Gualtieri et al. (2021), through the literature review of the research challenges on ergonomics and safety in industrial human–robot collaboration, pointed out the lack of studies on ergonomics compared to safety-related topics. Few studies were concerned with occupational health and indicated the benefits of human–robot collaboration (Cherubini et al., 2016; Brun and Wioland, 2021).

Numerous authors believed that collaborative robots contributed to the improvement of working conditions, productivity, MSD reduction (Sadrfaridpour et al., 2016; Awad et al., 2017; Pearce et al., 2018; El Makrini et al., 2019; Zanchettin et al., 2019; Gualtieri et al., 2020; Liau and Ryu, 2020; Palomba et al., 2021), improve the overall mental wellbeing of human operators (Parra et al., 2020), and minimize the time of execution the working activities (Hawkins et al., 2013). Ender et al. (2019) pointed out the relationship between human–robot collaboration and ergonomics (physical, cognitive, and organizational).

A review and detailed analysis of scientific research articles showed that the research on workers' effectiveness and manual and repetitive assembly work performance was mainly based on the determination of the correct body position (Fish et al., 1997; Leider et al., 2015). In scientific research, much less attention was paid to cognitive and perceptual factors that cause errors during the implementation of the work tasks (Fish et al., 1997). Falck and Rosenqvist (2012) showed that cognitive requirements are related to the operator's workload and errors made during the performance of the activities. Earlier research on mental and cognitive aspects relies on theoretical assumptions characterized by subjectivity (Parasuraman, 2003). The results obtained from the application of these methods are unreliable and biased (Parasuraman and Rizzo, 2006; Lehto and Landry, 2012).

Some authors pointed out the advantages of using EEG (Gevins and Smith, 2006) in measuring continuous and objective brain activity and the cognitive state of the operator (Luck et al., 2000; Murata et al., 2005; Jagannath and Balasubramanian, 2014) at the workplaces that require a high concentration of workers (such as assembly activities). The benefits of using an EEG device are based on the timely and objective detection in case of a drop in the attention and concentration levels, number of errors made, and so on. EEG systems provide the possibility of continual and objective measurement of workers' attention (Mijović et al., 2015, 2016a, 2017).

The literature review determined that a few scientific research articles have been written about physical and cognitive ergonomics within the human–robot collaboration, and there is room for further research in this area. Specific authors were engaged in the research of cognitive ergonomy in human–robot interaction (Maurice et al., 2013; Kim et al., 2018, 2019; Pearce et al., 2018; Lorenzini et al., 2019; Zanchettin et al., 2019; Gualtieri et al., 2020; Hopko et al., 2021) and some authors focused on the relationship between physical ergonomics and human–robot collaboration (Charalambous et al., 2016; Sadrfaridpour et al., 2016; Rossato et al., 2021).

Our study points out a wide range of experimental possibilities in human–robotic interaction. A modular and adaptive experimental set-up presented in an article will allow the researchers and practitioners to conduct neuroergonomic research seeking answers about workers' physical, mental, and emotional overload, fatigue, and decreased concentration. These aspects have become key indicators of product quality, including the constant problems with workers' absenteeism in the industry.

## Methods and Materials

This article presents a new, modular, and adaptive laboratory model of industrial assembly workstation (hereinafter referred to as workstation). This workstation model enables the realistic replication of assembly work activities in the industry, from simple ones to the complex interaction of workers and collaborative robots. During the design and construction of the laboratory model of the industrial assembly workstation, special attention was paid to the workspace for handling materials, parts, and components, considering that the operators should predominantly perform tasks within the golden zone. This zone is an ideal working area, where movements, reaching materials, stretching, and bending are minimized, and workers achieve the highest efficiency and productivity. The golden zone rules improve workplace organization and reduce muscle efforts and the occurrence of occupational diseases (MSDs). The workstations' construction is made of aluminum profiles (frames 40 × 40 mm and 40 × 80 mm), primarily used in the industry. The aluminum profiles are tightened with associated tensioning elements to stiffen the whole structure to give stability. The working surface is made of gray particleboard core covered with a silicone tablecloth protecting the piece from slipping during assembly.

Prolonged work in the same position causes strain on the operator's muscles, developing in the long-term occurrence of MSDs. Therefore, whenever working activities allow, operators should move from a sitting position to a standing position. Numerous studies have shown that back pain occurs in the workers who perform activities in a standing position (Andersen et al., 2007; Roelen et al., 2008; Nelson-Wong and Callaghan, 2010) over a long time, and therefore, operators must be allowed to perform activities by a combination of sitting and standing positions. The developed workstation is electrically height-adjustable using dual-lifting telescope system columns controlled by a 2-key hand switch and adapted to the anthropological characteristics of the participants. After a review of scientific research articles, it could be concluded that the best option would be for workers to perform activities on flexible workstations that are adjustable in height (Wilks et al., 2006). Also, the industrial work chair is height-adjustable, made of robust material, and characterized by stability when changing the participants' weight.

The workstation is upgraded with additional systems to fully simulate complex conditions characteristic of a natural work environment and enable advanced testing of participants' behavior during manual assembly tasks. An industrial computer is integrated into this workstation to monitor and control the performance of various work tasks, process visualization, and communication with the operator *via* HMI devices. A touchscreen PC is connected to the system for task definition and stimulus application.

Furthermore, special attention is paid to lighting. Lighting is an indispensable factor in the ergonomic design of the assembly workstation. It is essential to provide even illumination of the work surface to avoid straining their eyes when performing work activities. Individual reflectors that create superimposed solid shadows can cause eye strain, and, as the result, there is fatigue and a drop in concentration. Homogeneous LED lighting has been installed on the new industrial workstation since it produces only soft shadows, putting less strain on the eyes. Additionally, we set up an audio 5.0 system to emulate the sounds of the industrial environment. Different industries could record different sounds and show a realistic work environment for different workplaces.

**Figure 1:**
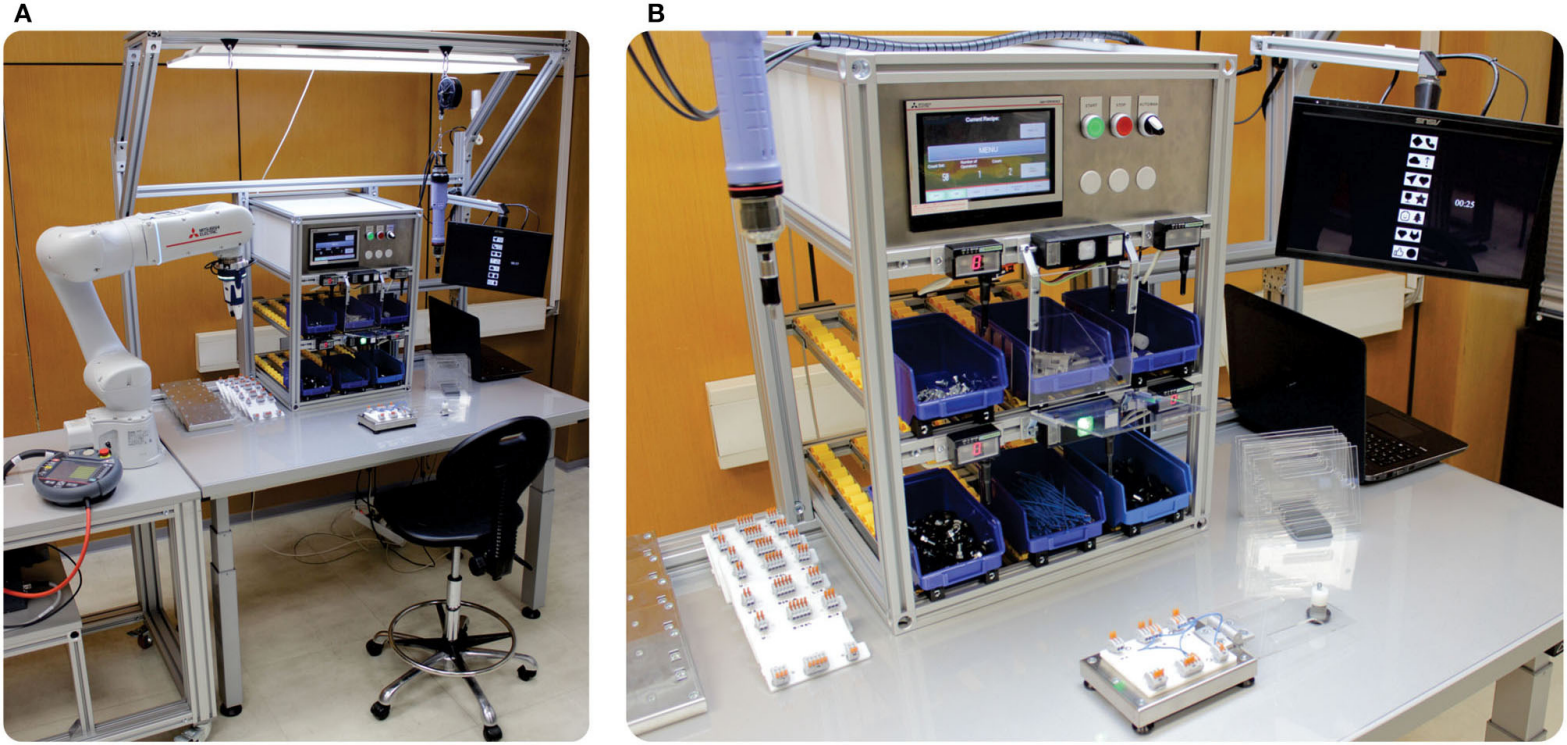
**(A,B)** A laboratory model of industrial assembly workstation.

The workstation (Figure 1A) is additionally equipped with blue plastic containers for storing assembly parts and tools, and the Poka–Yoke system for automatic control of assembly activities and prevention of errors. Systems that help workers to perform assembly activities make it easier to perform these activities and enable the worker to reduce errors (Fast-Berglund et al., 2013) and increase productivity (Hinrichsen and Bendzioch, 2018). The installed Poka–Yoke system (Figure 1B) has 6 independent lines to supply 6 different key components of the product, which are equipped with modules for access to the control at the entrance as well as the exit of the line. Vessels with mounting components move in a line *via* a wheeled conveyor. Poka–Yoke modules are equipped with indicator elements that indicate the next operation in the sequence and sensor elements to identify the fulfillment of individual orders. Removing the components for the current operation activates a sensor that automatically confirms the end of the current operation and gives a signal to activate the next operation.

Additional module for workstation represents a collaborative robot (cobot) station that enables the design of the work tasks where the operator and the robot will perform activities together. Unlike classic robots, cobots have built-in sensors that allow them to recognize and analyze workers' intentions and adapt their activities to the abilities of workers (Bonini et al., 2015) by monitoring the physical and cognitive workload of workers. The collaborative robot performs assembly activities that are monotonous, tiring, and repetitive or involve workers straining and bending. In this way, cobots improve working environment conditions by reducing worker workload as well as the risk of injuries at the workplace. Collaborative robots also perform those activities that require maximum precision and that operators cannot perform as reliably as robots. The operator performs activities that require a high level of knowledge and skills and decision-making skills (Figure 1).

**Figure 2:**
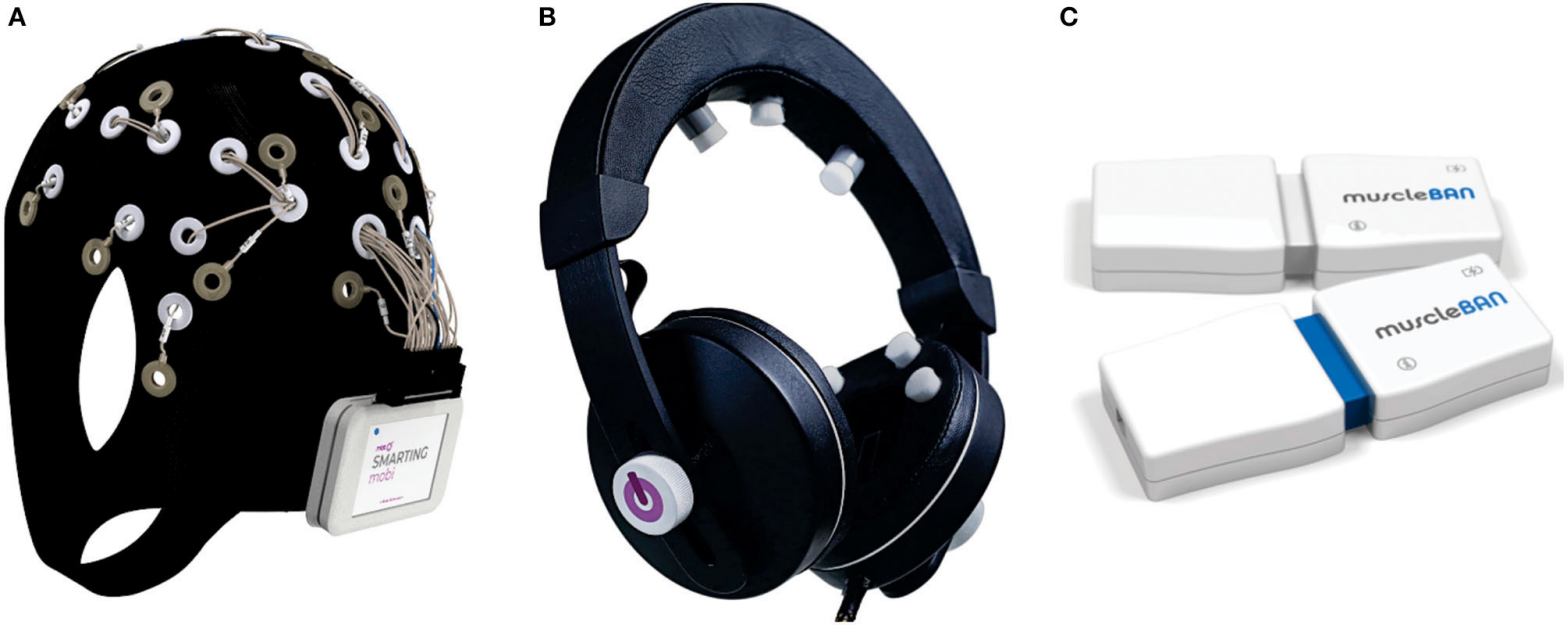
**(A)** EEG Cap, **(B)** EEG Smartfones, **(C)** EMG muscleBAN.

The innovative EEG system is used to design and conduct neuroergonomical experiments. Depending on the requirements of the experiments, EEG data could be acquired using the wireless EEG system in two possible configurations. The first one is using a 24-channel gel-based EEG cap (EASYCAP GmbH, Wörthsee, Germany) with 10–20 electrode placements (the Ag/AgCl electrodes) (Figure 2A). The EEG data are acquired using the lightweight EEG amplifier attached to the back of the cap. The Bluetooth connection is used as a communication protocol between the EEG amplifier and the computer (mBrainTrain, 2019). The second configuration uses the Smartfones (Figure 2B), the modified headphones to collect EEG data (mBrainTrain, 2019). The Smartfones use 4 gel-free electrodes placed around the ears and three in the central scalp zone (Kartali et al., 2019). The EEG data were acquired using a 500 Hz sampling frequency in both configurations. In the first configuration (the gel-based system is used), the electrode impedances were kept below 10 kΩ, whereas in the second configuration (the gel-free system), they were kept below 20 kΩ because of the different electrode properties. For EMG measurements during the neuroergonomical experiments, muscleBAN (PLUX Wireless Biosignals, Portugal) was used. This wearable, wireless (Bluetooth or Bluetooth Low Energy data transmission) device combines a single-channel EMG sensor, triaxial accelerometer, and magnetometer and, in that way, enables real-time acquisition with up to 16-bit resolution at up to 1,000 Hz sampling rate (Figure 2C). Small dimensions of the device and an internal battery that ensure the autonomy of 8 h make it suitable for workplace arm muscle activity and motion data monitoring when placed in pairs on both forearms.

**Figure 3:**
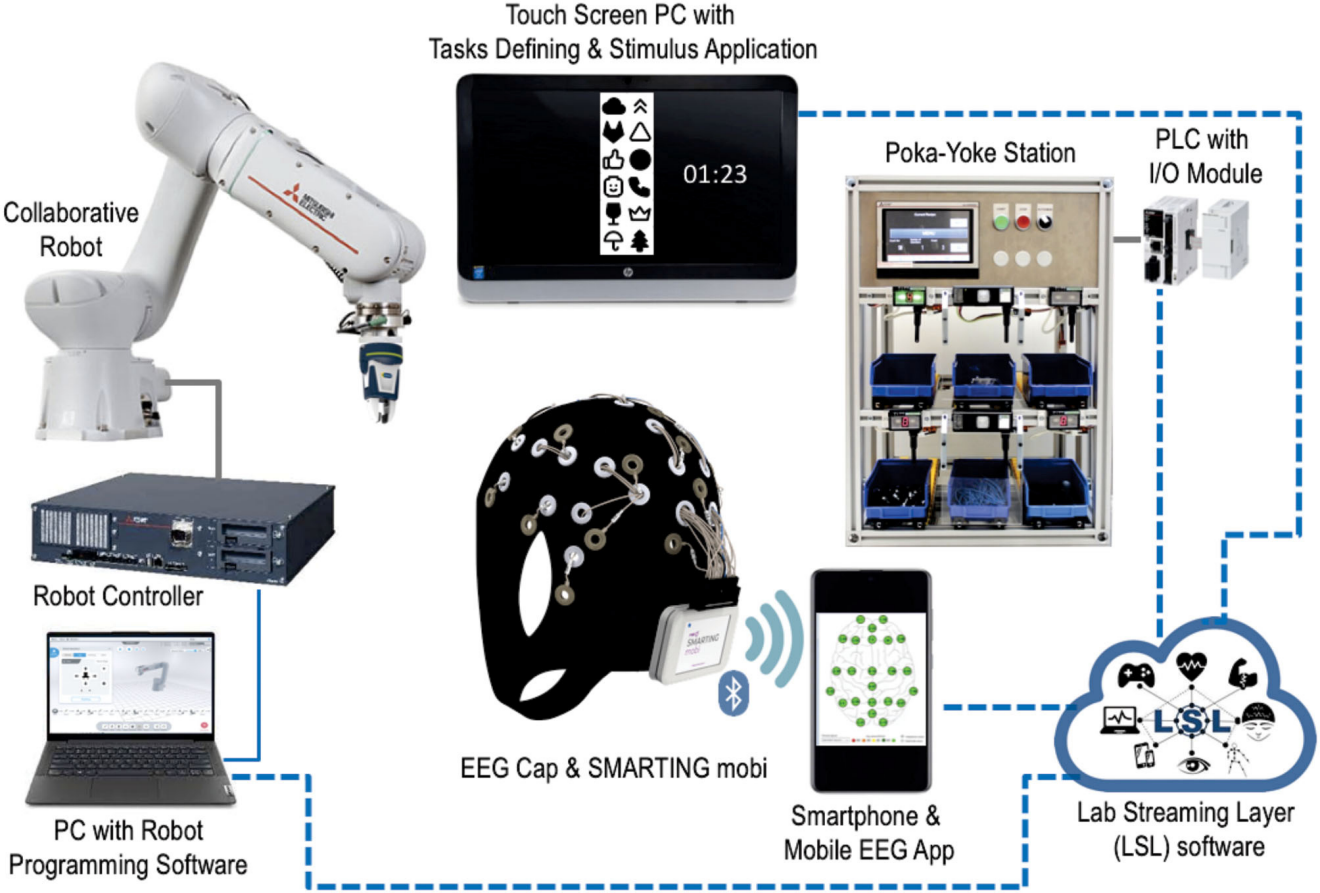
LSL integration of key measurement set-up elements.

One of the most demanding challenges in all experiments is the proper synchronization of all elements in the measurement set-up, which needs to ensure that the timing of all events and recorded data are defined and known with sufficient precision. If the timing of these events cannot be well-measured, this will cause the loss, reduction, or blurring of any measured data and their relations to trigger events. The function of synchronization is to eliminate timing errors, which cannot be eliminated on hardware and measurement set-up levels or to be corrected after analysis, so they must be solved before the measurement starts. For synchronization, a specific software/API package was used, called the Lab Streaming Layer (LSL), as a powerful tool that allows multiple continuous data streams and discrete marker timestamps to be acquired in an eXtensible Data Format (.XDF). The inputs from multiple devices, connected to one measurement set-up, are collected and synchronized *via* LAN network using LSL (Figure 3).

### Description of the Research Scenarios

The study of behavior and reactions during collaborative interaction between workers and cobot represents a particular challenge, where positive characteristics of the workers (adaptability, creativity, ability to make quick decisions, dexterity, perception, agility, cognitive abilities, ability to think critically, and intellectual abilities) are combined with technical characteristics of cobots (strength, endurance, precision, speed, repeatability, and consistency) (Helms et al., 2002; Kruger et al., 2009; Murashov et al., 2016) to perform work activities more efficiently and safely. On the other hand, in traditional work environments, work activities are strictly divided into those performed by robots and activities performed by workers (Wongphati et al., 2015; Maeda et al., 2016).

**Figure 4:**
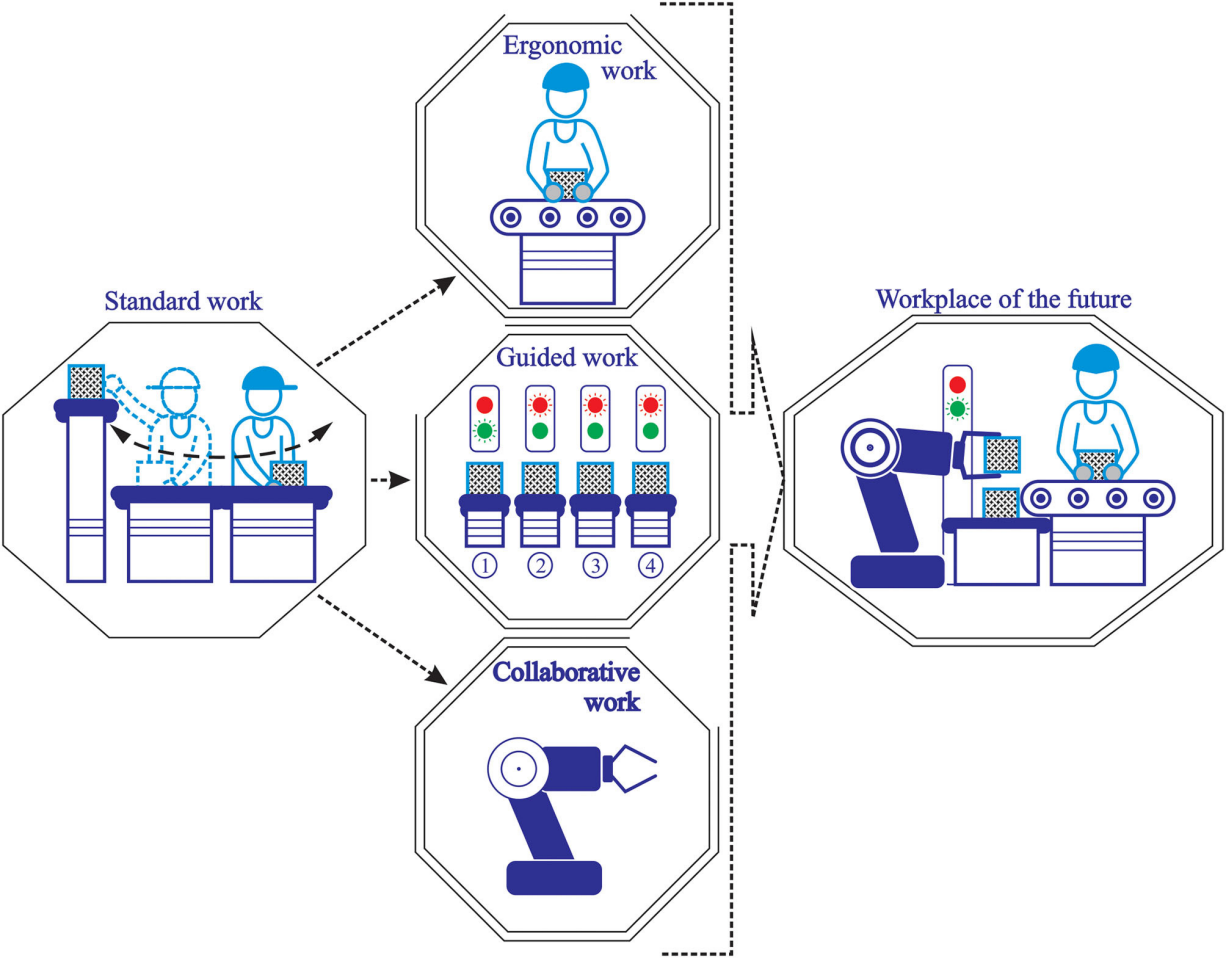
The research scenarios.

The designed workstation represents the laboratory infrastructure used for conducting neuroergonomic experiments and studying the behavior of operators at the workplace. Based on workstation construction and integrated elements, four basic scenarios could be performed to make workers' behavior comparative analyses (Figure 4):

1. **Standard work**—performing manual assembly work tasks for a complex product without any specific intervention or improvement at the workplace. Work is performed on workstation “as is” without personal adjustments according to ergonomic or “golden zone” standards.
2. **Ergonomic work**—work is performed on an ergonomically optimized workstation with a workplace organized in conformity with the ergonomic and “golden zone” principles and standards.
3. **Guided work**—participants perform the same work tasks as in the first scenario but with the additional involvement of the Poka–Yoke station. The Poka–Yoke system has a role in guiding operators through the repetitive process of assembling parts and components, from operation to operation, generating the start of each subsequent step in a predefined sequence of steps and thus preventing human errors.
4. **Collaborative work**—participants perform work tasks with the support of a collaborative robot, where the collaborative robot performs repetitive, simple activities that do not require thinking and decision making.

**Figure 5:**
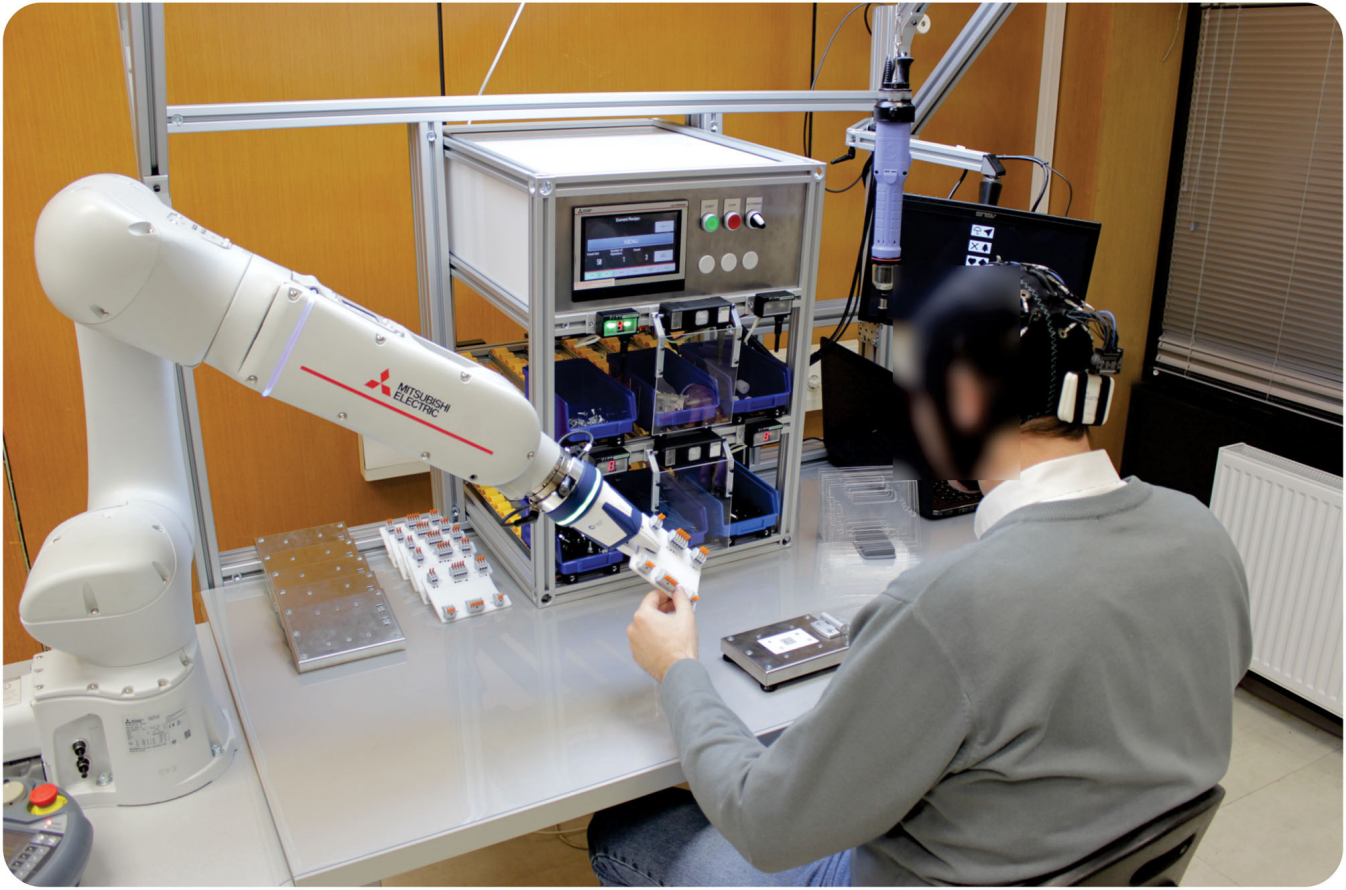
The workplace of the future.

Previously defined scenarios represent identified tools, methods, and techniques that could ensure the transformation and improvement of the standard industrial workplace, for manual assembly tasks, into the workplace of the future (Figure 5). All mentioned directions will be used in the nearest future, in some forms and combinations, and that is why continuous work and investigation of human behavior and reaction, to each of them, have significant importance.

### Description of the Experimental Session

The authors conducted a neuroergonomic study according to the first scenario (standard work) during the initial research phase. The participants' working tasks were manual, monotonous, and repetitive. Operator assembled parts and components into the final product following the order of assembly and pre-defined provisions concerning positioning the parts and components, and so on. The experiment was conducted in conditions that were, generally, in conformity with the natural industrial environment. The selected work activities met several prerequisites similar to actual industrial tasks, repeatable, and feasible in laboratory conditions. Activities and tasks that were identified as characteristic during the visits to the companies and interviews with persons responsible for production and safety were selected. This approach is called participatory ergonomics intervention (De Guimarães et al., 2015). In this way, the simulation of actual production is provided without changing the structure of components and assembled parts.

To perform the experiment, the authors developed and constructed a prototype model of an industrial product, which is an abstraction of the connection plate and consists of a metal base made of steel sheet with built-in threaded elements and a transparent acrylic cover connected with an aluminum hinge (combination of three materials). Adjustable legs and electrical connectors of various sizes are placed on the stand. Wiring and connection of electrical connectors can be reported in several ways (different job variation options). The product can be completely disassembled, an essential factor for performing multiple experiments. The very fact that such research can be conducted in replicated work environments, where the work process is simulated, is an excellent progress, and it can bring necessary knowledge about worker cognition, which can later be used in designing specific jobs (Mijović et al., 2016b).

**Figure 6:**
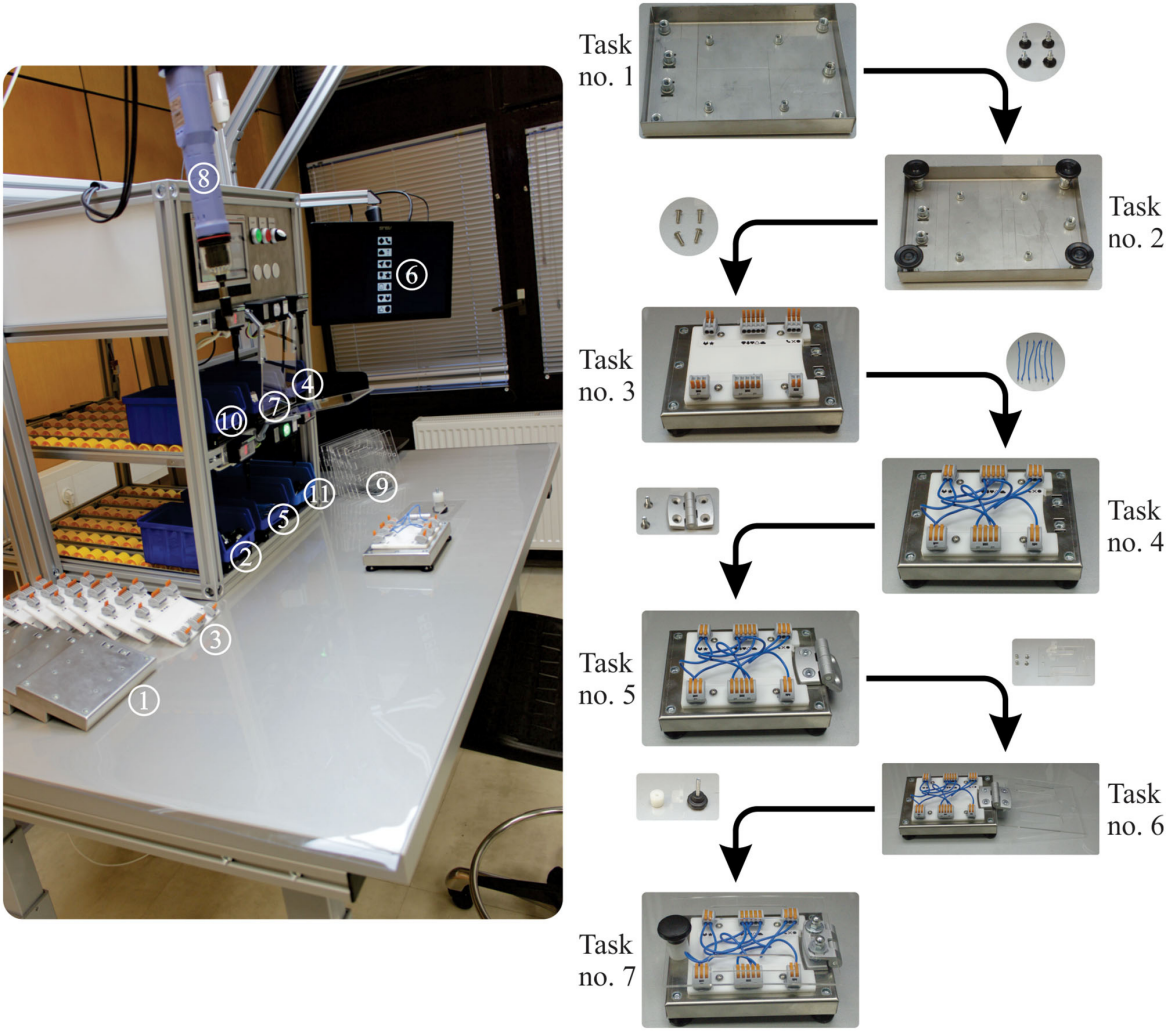
Description of the experimental session.

Before starting, the entire experiment and its purpose are explained to the participant. EEG cap is mounted on the participant's head, and the EEG device and associated computer are configured and set according to the internal protocol. After the final check is done, the technician starts the EEG device and plays relaxing music for 5 min. After 5 min, the participant starts the assembly process. The whole experiment consists of two rounds per 60 min, each, with a 15 min break between the sessions. The product assembly takes approximately 4 min. The assembly tasks and the components and tools used (①– ⑪) are shown in Figure 6.

**Figure 7:**
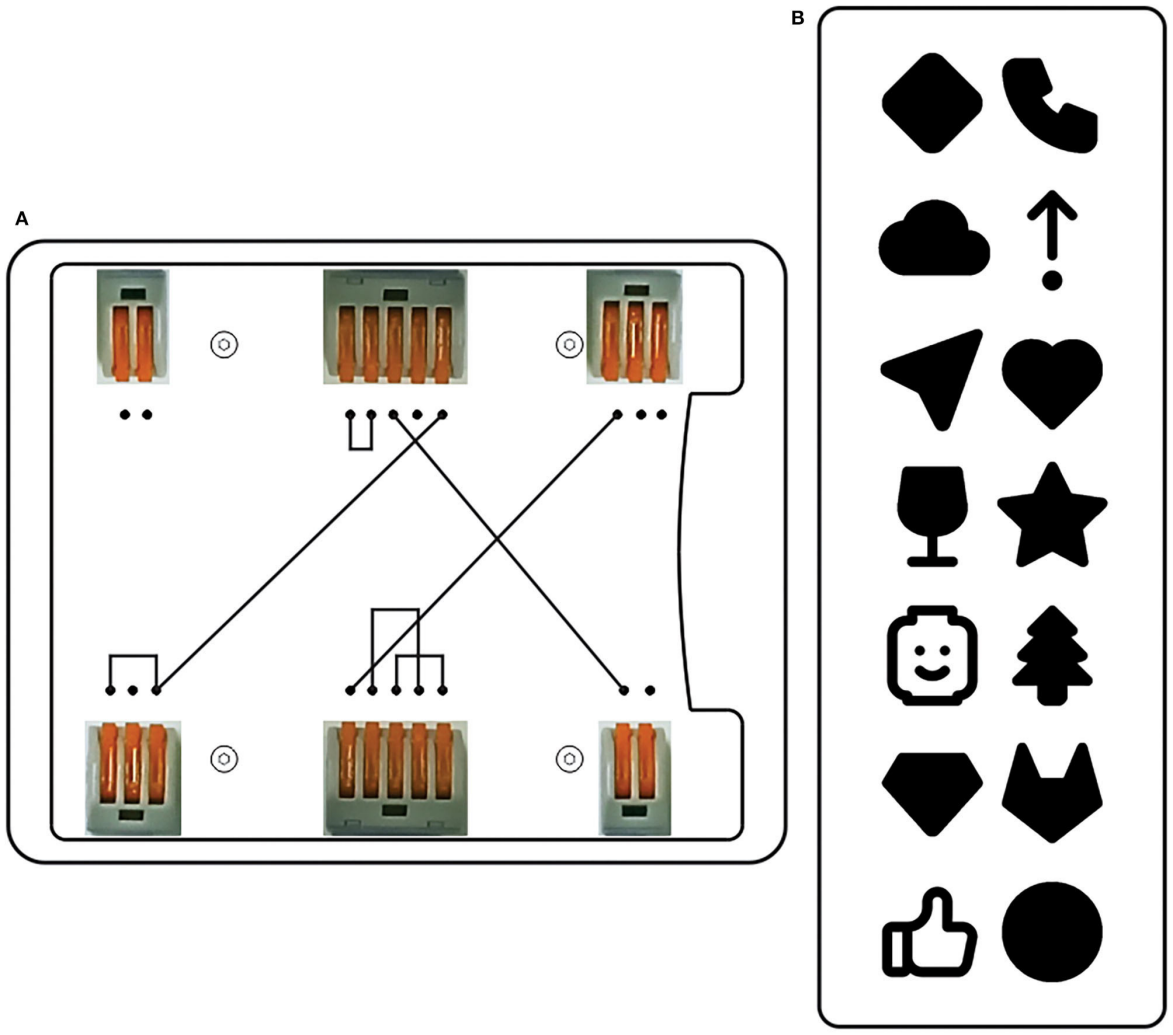
Types of schemes **(A)** easy to connect, **(B)** harder to connect.

**Task no. 1:** Take the steel plate base from the lot ① and place it in the appropriate place, in an upside-down position.
**Task no. 2:** Take supports (four pieces) from the container ② and tighten them to the end, manually, in their positions.
**Task no. 3:** Turn the object to the upper side. Take the white acrylic with prepared glued connection elements from the lot ③. Take four round hex screws (M4x16) from container ④ and tighten them with an adequate hex key wrench.
**Task no. 4:** Take seven, one-by-one, wires from a container ⑤ (wires are 150 mm in length and prepared for connection) and connect them. The connections (number and task definition) are carried out according to the information showed on the installed touchscreen PC ⑥. There were two types of prepared wiring schemes. The first type was schemes assumed to be easy to connect. The second type was assumed to be challenging to connect. The participant did not know which order scheme would appear on the monitor. The participant randomly gets a picture or pair of the symbols that have to be connected (Figure 7).
**Task no. 5:** Take one hinge from container ⑦, two countersunk screws (M6x12), and tighten them with the adjustable torque screwdriver ⑧ hung on the balancer.
**Task no. 6:** Take one transparent acrylic from plot ⑨, two countersunk screws (M6x12), and two cap nuts to fasten a hinge and the acrylic.
**Task no. 7:** Take one cylindrical plastic roller from container ⑩ and one threaded spindle rod from container ⑪ to tighten the transparent acrylic to the steel plate base.

Finished prototype model of an industrial product is stored in the predefined place while the participant starts with task no. 1 again.

### Initial Results and Discussion

**Figure 8:**
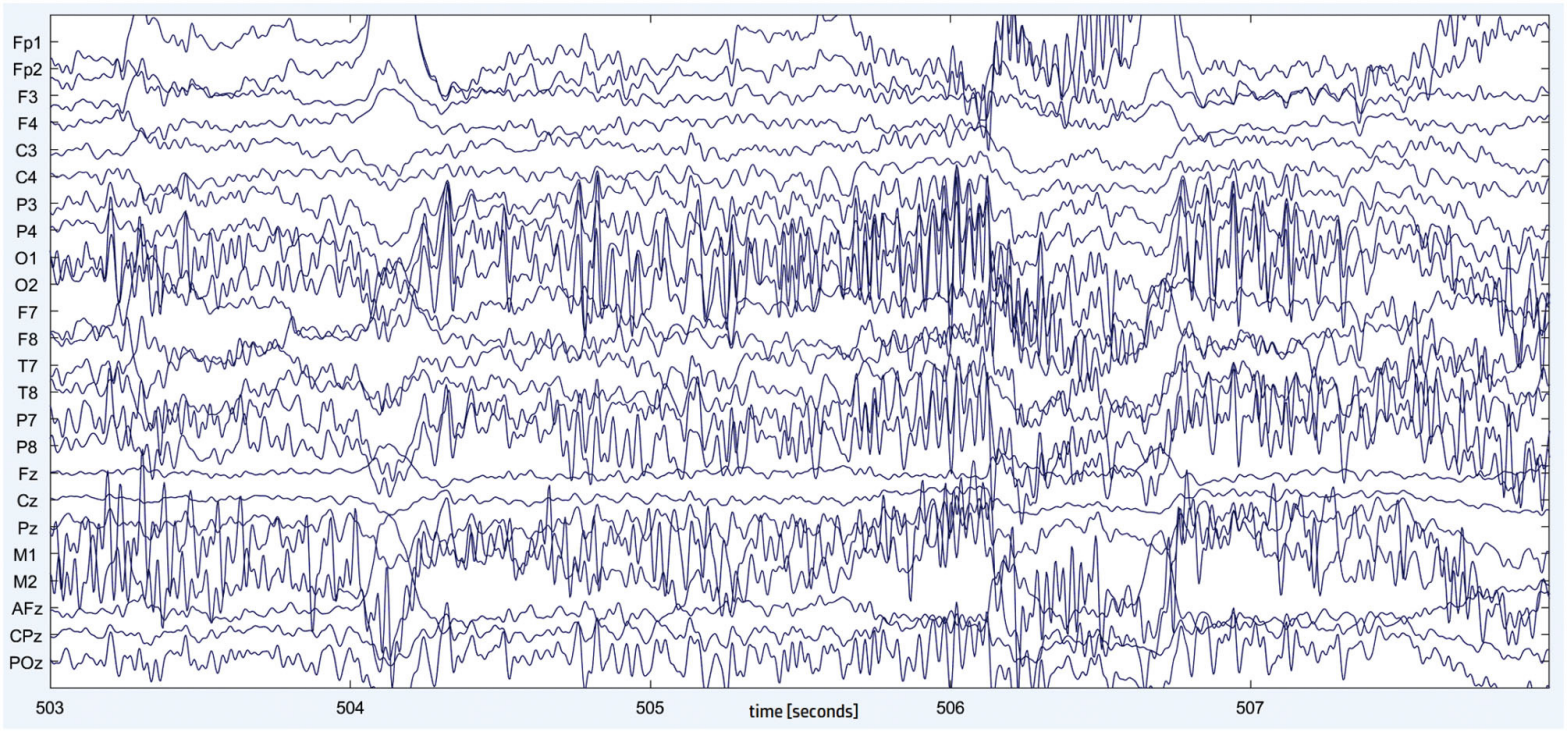
Band-pass filtered 24-channel EEG signal (5 s) recorded during the assembly task. On the *y*-axis we have signals from 24 electrodes by their name.

The study's main idea was to propose, develop, and test a modular and adaptive laboratory workstation model that could be used for various types of experiments requested by the industry. The initial results are related only to examining the possibility of conducting experiments on a developed workstation and whether it is possible to obtain satisfactory initial results by imitating the working environment. Collected EEG data were processed using MATLAB (MathWorks, Massachusetts, United States) and EEGLAB (https://sccn.ucsd.edu/eeglab/index.php). The EEG signals were first band-pass filtered in the range 1−40 Hz, using an FIR filter generated by the EEGLAB. The amplitude of the signal is in the range from 1 to 100 μV (Figure 8).

**Figure 9:**
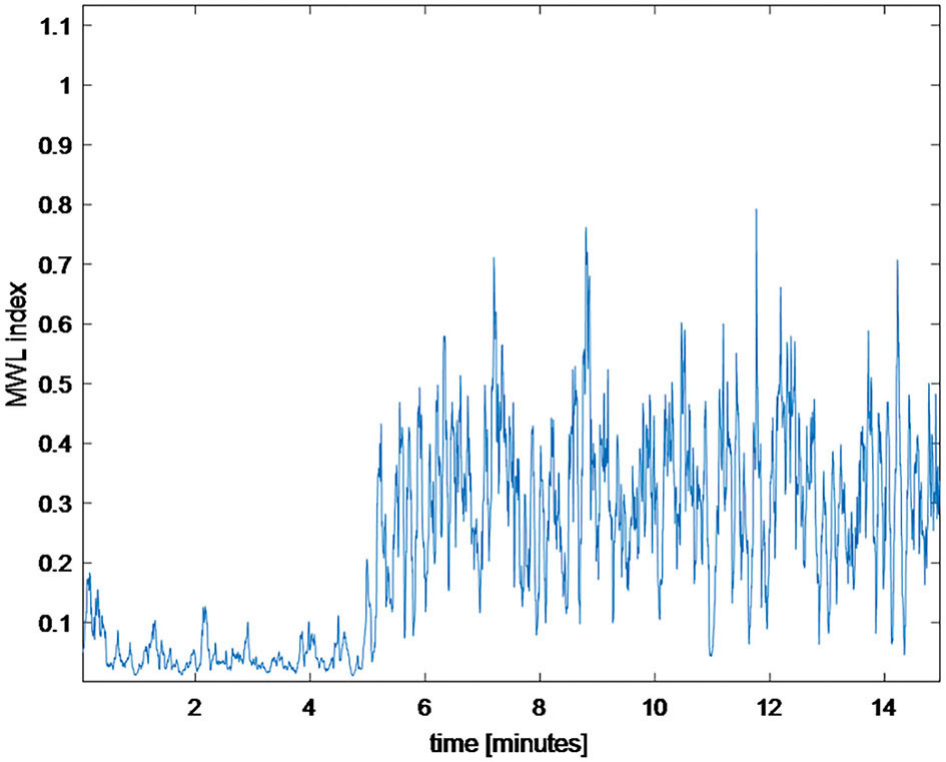
MWL index of the first 15 min of the experiment (window size is 5 s).

Research has shown that, in response to the mental demands of the task being performed, EEG signals tend to change predictably, more specifically that EEG spectral power correlates with task complexity (Brookings et al., 1996; Gevins et al., 1997; Stipacek et al., 2003; Missonnier et al., 2006). Namely, due to observable changes in frontal midline theta band (4–7 Hz) and parietal midline alpha band (8–12 Hz), their ratio can be employed to estimate MWL (Holm et al., 2009; Zhang et al., 2018; Andreessen et al., 2021). The so-called MWL index is obtained by computing the ratio between signal power in the theta band (4–7 Hz) from the frontal midline electrode (Fz) and signal power in the alpha band (8–12 Hz) from the parietal midline electrode (Pz). We windowed the raw signal to compute the MWL index (using 5 s windows with 4.9 s overlapping). The metric can be seen in Figure 9. During the first 5 min, a subject was idle (listening to some relaxing music) while he was involved in the assembly work for the rest of the time. As we can see, this is evident from Figure 8, as the respective MWL index was low for the first 5 min.

Two participants took part in the initial experiment on the developed modular and adaptive laboratory set-up. We can extract comparative statistics for the first session to prove that the MWL index is lower for lower-engagement activity (the first 5 min of resting time). The statistical data are shown in Table 1.

**Table 1:** Average MWL indexes for both subjects in the cases of resting and active time.

	**Participant no. 1**	**Participant no. 2**
Resting MWL index	0.0472	0.1737
Active MWL index	0.3013	0.9187

The statistics prove that MWL is lower during the first 5 min of the session while subjects are taking rest. In addition to that, note that EEG signal has different strengths (amplitudes) for different participants, as the result of significantly different MWL indexes for participant no. 1 and participant no. 2 under the same task difficulty level. This is why EEG is usually normalized when processing signals of different participants together. One of the purposes of this experiment was to distinguish periods of low and high complexity schemes and estimate task difficulty with regard to time by looking at the MWL index in real time. However, we would need to test and record more subjects to conduct that analysis. That way, we could make an average over their normalized MWL index vs. time graphs and resolve the problem of individual differences between subjects. The result would be an objective (not participant-dependent) task difficulty with regard to time. We plan to carry out this research soon and on a larger sample. This research explains that it is possible to conduct neuroergonomic research on a new, modular, and adaptive laboratory workstation model.

We also noted some technical difficulties during the experiment. One of the issues was switches stiffness. Participants experienced problems if they made a wrong connection with a wire and had to unlock the switch and lock it again. We plan to solve the same making more ergonomic schemes. Another problem was that the chair was inappropriate for the experiment this long, as both subjects confirmed that they felt mild pain in their backs after some time of being in that position. Furthermore, the main participants' remark was losing focus during the 3-h experiment. They concluded that it is possible to lose focus very easily, which could be the new research hypothesis for future research work.

In the future, during our research activities, we will continue to collect data corresponding to the remaining three different scenarios (ergonomic work, guided work, and collaborative work). This should enable a comparative analysis of participants' behavior and monitor the operators' psychological reactions during the implementation of the same or similar work tasks under different scenarios. The most important part of the planned research activities is related to assessing the neuroergonomics parameters and examining operators' reactions during the performance of the working activities in cooperation with the collaborative robot.

## Conclusions

Workplaces with high repetitiveness of tasks, high noise levels, and poor ergonomics can cause both mental and physical stress and reduce the operator's attention. Over time, products with many or similar components can cause an increase in the number of errors. The increasing variety of products was also identified as the leading cause of the complexity perceived by an operator in carrying out his tasks (Olwal et al., 2008). Taking into consideration that the workforce is getting older, it is necessary to pay attention to, so far, not so attractive parameters for monitoring and improvement such as wellbeing, operators' satisfaction, attention, concentration, and fatigue. To enable monitoring of these parameters, a new, modular, and adaptive laboratory model of industrial assembly workstation for conducting advanced research in the field of ergonomics, neuroergonomics, and human–robot interaction is designed and built. This recently designed workstation eliminates all limitations that characterize a traditional workstation.

This newly developed workstation is designed to be operator-centered and thoroughly adapted to the operator's needs, abilities, and limitations. The anthropometric characteristics of the workers were taken into account so that the workstation is suitable for both males and females and so that the workers can carry out assembly activities within the golden zone. This workstation includes the assembly area and it has a built-in Poka–Yoke system. It can guide the actions carried out by the worker and aims to improve the quality of the product being assembled. Furthermore, it minimizes errors accidentally made by the operators due to a drop in the concentration and intentional errors.

The main elements from the industry were replicated in the laboratory, taking into consideration spatial dimensions of the workplace and ambient conditions. This article describes an innovative neuroergonomic experimental set-up studying operators' comparative habits and behavior at the workplace for four different scenarios—standard work, ergonomic work, guided work, and collaborative work. This ensures the transformation and improvement of the standard industrial workplace into the workplace of the future. The assembly task proposed by the authors consists of the developed and constructed prototype model of an industrial product that can be disassembled and thus used in numerous experiments. Participants in the laboratory examination carry out characteristic and standardized assembly activities. Initial neuroergonomic tests using an EEG device were conducted to show various research possibilities on the workstation. In a replicated workplace, the whole process of producing the final product was simulated. Operators' reactions, behavior, and responses to sophisticated conditions in the work environment are monitored. The preliminary experiments showed that it is possible to conduct neuroergonomic research on a new, modular, and adaptive laboratory model of industrial assembly workstation. Moreover, the industry could request various scenarios to improve the operators' ergonomics. The requested scenario will be adapted in the advanced laboratory set-up, then tested and analyzed with specific outputs proposed to solve the identified problem.

The experimental set-up presented in this article is the basis for conducting advanced research in the future. We will collect data regarding ergonomic, guided, and collaborative work that will show participants' behavior and psychological reactions during the implementation of the same or similar work tasks. These results will be analyzed through a comparative analysis to define which parameters are most important to be monitored. The main focus will be on examining operators' reactions during working activities in cooperation with the collaborative robot.

## Data Availability Statement

The original contributions presented in the study are included in the article/supplementary material, further inquiries can be directed to the corresponding author/s.

## Ethics Statement

The studies involving human participants were reviewed and approved by Ethics Committee of the Faculty of Medical Sciences, University of Kragujevac Decision number: 01-6471, date: June 3rd, 2021, based on submitted study protocol no. 01-5578 from May 18th 2021. The patients/participants provided their written informed consent to participate in this study.

## Author Contributions

MS and IM conceive the idea and concept. MD and IM designed and built a workstation. MP, MD, and CC designed the experiment. CC and AV collected data. MP and AV analyzed data. MS, MD, and IM wrote the manuscript. All authors read and approved the final manuscript.

## Funding

This study was partially funded by the Innovation Fund Republic of Serbia, StayAlert—A New Tool for Safe Work (Project ID: 50231). This study was partially funded by the European Commission, HORIZON 2020 Marie Curie Training Network Collaborative Intelligence for Safety Critical systems (Grant agreement ID: 955901).

## Conflict of Interest

MP is employed by mBrainTrain d.o.o., Belgrade, Serbia. The remaining authors declare that the research was conducted in the absence of any commercial or financial relationships that could be construed as a potential conflict of interest.

## Publisher's Note

All claims expressed in this article are solely those of the authors and do not necessarily represent those of their affiliated organizations, or those of the publisher, the editors and the reviewers. Any product that may be evaluated in this article, or claim that may be made by its manufacturer, is not guaranteed or endorsed by the publisher.
